# A Practice Pathway for the Treatment of Night Wakings in Children with Autism Spectrum Disorder

**DOI:** 10.1007/s10803-023-06026-2

**Published:** 2023-06-26

**Authors:** Anjalee W. Galion, Justin G. Farmer, Heidi V. Connolly, Virginia D. Allhusen, Amanda Bennett, Daniel L. Coury, Janet Lam, Ann M. Neumeyer, Kristin Sohl, Manisha Witmans, Beth A. Malow, Autism Treatment Network/Autism Intervention Research Network on Physical Health (ATN/AIR-P) Sleep Committee

**Affiliations:** 1 Division of Neurology, Children’s Hospital of Orange County, 1201 W. La Veta Avenue, Orange, CA 92868 USA; 2 Massachusetts General Hospital for Children, Boston, MA USA; 3 University of Rochester, Rochester, NY USA; 4 Children’s Hospital of Philadelphia, Philadelphia, PA USA; 5 Nationwide Children’s Hospital, Columbus, OH USA; 6 Kennedy Krieger Institute, Baltimore, MD USA; 7 Harvard Medical School, Boston, MA USA; 8 University of Missouri School of Medicine, Columbia, MO USA; 9 University of Alberta, Edmonton, AB Canada; 10 Vanderbilt University Medical Center, Nashville, TN USA

**Keywords:** Sleep problems, Autism spectrum disorder, Night wakings, Treatment for insomnia, Sleep initiation, Sleep maintenance

## Abstract

Children with autism spectrum disorder (ASD) report high rates of sleep problems. In 2012, the Autism Treatment Network/ Autism Intervention Research Network on Physical Health (ATN/AIR-P) Sleep Committee developed a pathway to address these concerns. Since its publication, ATN/AIR-P clinicians and parents have identified night wakings as a refractory problem unaddressed by the pathway. We reviewed the existing literature and identified 76 scholarly articles that provided data on night waking in children with ASD. Based on the available literature, we propose an updated practice pathway to identify and treat night wakings in children with ASD.

## Introduction

Approximately 1 in 44 children have autism spectrum disorder (ASD), defined by diagnostic criteria that include deficits in social communication and social interaction, and restricted or repetitive patterns of behavior, interests, or activities (Maenner et al., 2021). Sleep problems commonly co-occur in this population; indeed, sleep problems are estimated to be more than twice as common in young children ages 2–5 years with ASD than in the general population (Reynolds et al., 2019), and they often persist into adolescence (Goldman et al., 2012). Approximately 50–80% of parents of children with ASD report sleep problems in their child (Couturier et al., 2005; Goldman et al., 2012; Krakowiak et al., 2008), including various types of insomnia, such as difficulty falling asleep, bedtime resistance, prolonged night wakings, and short sleep duration (Richdale & Schrek, 2009; Williams et al., 2004).

Sleep problems in children with ASD are associated with hyperactive/impulsive behavior, disruptive behavior and other daytime behavior problems. Night wakings in particular show associations with physical aggression, irritability, and hyperactivity (Mazurek & Sohl, 2016). Night wakings and sleep onset delay contribute to short sleep duration, which is associated with poorer adaptive functioning in daily living skills, social skills, motor development; and restricted and repetitive behaviors (Taylor et al., 2012; Veatch et al., 2017). Health-related quality of life is also affected in children with ASD and short sleep duration (Delahaye et al., 2014). Furthermore, bidirectional relationships of disordered sleep with immune dysregulation make children at greater risk for other physical and neuropsychiatric problems (Iranzo, 2020; Louveau et al., 2015; Winkelman & Lecea, 2020; Yin et al., 2021; Zielinski & Gibbons, 2022). Beyond the child’s own health, consistent sleep problems contribute to maternal stress and poorer maternal mental health (Hodge et al., 2013). Fortunately, treatment of sleep problems in children with ASD, using behavioral or pharmacologic approaches, results in improvements in child behavior and family functioning (Malow et al., 2012, 2014).

To address the multifaceted problem of sleep disturbance and related impairments, the joint Autism Speaks Autism Treatment Network and Autism Intervention Research Network on Physical Health (ATN/AIR-P) published a practice pathway for the identification, evaluation, and management of insomnia in children with ASD (Malow et al., 2012). This practice pathway was based on expert consensus with the goal of capturing best practices for an overarching approach to insomnia by a general pediatrician, primary care provider, or autism medical specialist, and included a systematic literature review and grading of evidence. Key points of the practice pathway included (a) screening the child for insomnia using a few targeted questions related to key sleep concerns (e.g., sleep onset delay, co-sleeping, sleep duration, night wakings); (b) identifying and managing medical contributors that can affect sleep (e.g., obstructive sleep apnea, gastroesophageal reflux, epileptic seizures, atopic disease such as asthma and eczema), (c) providing educational/behavioral interventions, and (d) close follow-up, with institution of medications or referral to a sleep specialist for persistent sleep problems. The practice pathway was designed to be broad and cover a variety of sleep problems.

Network providers and parents noted that the 2012 pathway mainly addressed difficulties with sleep onset. The separate issue of night wakings remains challenging and continues to have clinical significance. Even after controlling for the effects of age and sex, night wakings have shown a strong association with daytime behavioral problems (Mazurek & Sohl, 2016). The prevalence of occasional or frequent night wakings in an ATN-related sample was approximately 50% (Katz et al, 2018). There are several reasons for emphasizing night wakings. Night wakings may be reflective of specific medical problems, such as obstructive sleep apnea, which wakes children from sleep to promote a resumption of breathing, poorly controlled asthma or atopic disease, all of which can result in sleep disruption. Indeed, a wide range of major medical conditions such as gastroesophageal reflux (GERD), constipation, restless leg syndrome, or seizures can be associated with night wakings. Finally, although educational/behavioral approaches and pharmacologic intervention are often effective in addressing sleep onset delay, bedtime resistance, and co-sleeping (when reported as problematic by the parent or caregiver), in our clinical experience the sleep concern of night wakings is often refractory to treatment with educational/behavioral approaches and/or pharmacologic intervention. Therefore, the ATN/AIR-P Sleep Committee reconvened to update the 2012 practice pathway for insomnia, with an emphasis on night wakings. In this review, the committee examined the definition of night wakings including number, duration and impacts on children, families and caregivers. The committee recognized that a working definition for night wakings would improve differential diagnosis and evaluation of clinical interventions.

## Methods

### Systematic Review of the Literature

A systematic literature review was conducted to find evidence regarding the treatment of insomnia in children diagnosed with ASD (questions and search terms available on request from the authors). Consistent with the International Classification of Sleep Disorders 3rd edition (2014) diagnostic criteria for insomnia, we included difficulty initiating sleep, difficulty maintaining sleep, and waking up earlier than desired. The reviews were completed by nine physicians and one research coordinator. Each physician reviewed 20–25 research abstracts, five of which were double reviewed by a second reviewer. Reviewers indicated whether an article was “definitely relevant,” “possibly relevant,” or “not relevant” to the issue of insomnia in children with ASD. Articles considered definitely relevant were advanced for full text review (review form is included as an end note). Those considered “ not relevant” by all reviewers were excluded from further consideration. The remaining articles (i.e., those with “possibly relevant” or conflicting characterizations) were reviewed by AG and JF.

Search engines included PubMed, OVID, CINAHL, PsycINFO, EBM Database of Abstracts and Reviews of Effects, and the Cochrane Database of Systematic reviews. The search was limited to studies with human subjects conducted between 1/1/1990 and 10/15/2018. For the most comprehensive evaluation of the literature on the treatment of night wakings, studies published prior to the 2012 practice pathway were included if data were not presented in that prior pathway. This initial search yielded 981 manuscripts. Other systematic literature reviews found in this initial search were reviewed for primary manuscripts not captured in the search. This resulted in an additional 13 manuscripts. After removing the systematic reviews and any duplicate manuscripts, 583 unique abstracts remained. These were each reviewed by two ATN/AIR-P Sleep Committee members. Reviewers identified 212 manuscripts for full text review, with 76 articles providing data on night wakings. Due to the paucity of data on night wakings, we did not set specific exclusion criteria based on sample size.

The 76 resultant articles were each evaluated using the Grades of Recommendation, Assessment, Development and Evaluation (GRADE) for Systematic Reviews guidelines (Guyatt et al, 2011). The articles were distributed among ten authors for review. Each author presented their findings to the full ATN/AIR-P Sleep Committee in monthly meetings, and questions were resolved by committee consensus.

One of the challenges in our evaluation and understanding of night wakings was the lack of a consistent definition or reporting format. Of the 76 papers reviewed the most common tool used for reporting of night wakings was parental report, via sleep diary and/or sleep survey. A small subset of articles relied upon polysomnography or actigraphy (instead of or in addition to parent report measures). A variety of surveys were used across these studies to measure sleep disturbances, including the Basic Nordic Sleep Questionnaire (BNSQ), Simonds & Parraga Sleep Questionnaire (SPSQ), Children’s Sleep Habits Questionnaire (CSHQ), Epworth Sleepiness Scale (ESS), and other locally developed sleep questionnaires. Although not all instruments in the set of 76 articles were available for committee review, most available questionnaires included one or two items on the occurrence of night wakings.

## Results

Across the 76 articles reviewed, there was no consistent definition for night wakings. Most articles (63/76) relied upon parent reported measures to assess night wakings..About one-third of the reports (27/76) included actigraphy. Polysomnography was less frequently used (10/76)., or retrospective clinical observation by chart review of parent reported sleep duration or completed sleep questionaires. Within the articles, several sleep disturbances were reported that the committee felt qualified as night wakings. These included nighttime or early morning wakings by subjective parent report, as well as decreased sleep efficiency, sleep fragmentation, and increased wake after sleep onset (WASO) as measured by polysomnography (PSG).

The 76 studies were placed into one or more of five broad categories based on the focus of the article: 1) frequency of night wakings, 2) frequency of night waking in children with ASD vs. children without ASD, 3) evaluation of tools used to identify night wakings, 4) other factors or conditions associated with night wakings, and 5) treatment/intervention for night wakings. Articles that addressed multiple domains were repeated in all relevant categories.

### Frequency of Night Wakings in Children with Autism Spectrum Disorder

The prevalence of night wakings is difficult to accurately identify due to a lack of a consistent measure; however, our review supports the contention that night wakings are a common concern in the pediatric ASD population. The most common sleep disturbances reported, apart from increased night wakings, were increased wake after sleep onset (WASO), poor sleep quality, and decreased sleep efficiency. These were identified predominantly by polysomnography and actigraphy, though with some subjective identification of poor sleep quality on questionnaires. Most articles identified some difficulty initiating or maintaining sleep (DIMS) but did not necessarily specify the type of sleep disturbance. Of note, there were limited studies that evaluated night wakings alone; most of the articles we reviewed evaluated night wakings as part of a constellation of broader sleep disturbances.

Table 1 shows the nine papers that provided a calculated rate of night wakings in children with ASD without a comparison group. These studies indicate that the prevalence of night wakings in children with ASD ranges from 0 to 84% depending on the definition used and the method of reporting and the population studied. Based on the relatively small number of available studies, there is insufficient data to pinpoint an accurate range for the prevalence of night wakings in children with ASD. However, the study with the largest number of subjects (n = 210) reported a prevalence of 34% (Williams et al., 2004). This seems to be a reasonable estimate of the prevalence of night wakings in children with ASD given that it utilized the largest sample size, it represents the median rate reported across these studies, and it is consistent with the literature on sleep disturbances in children with Autism (Malow et al., 2016). One study that may be excluded from our evaluation is that of Oyane and Bjorvatn (2005) as it studied older teens and adults ranging from 15 to 25 years with a sample size of 15 participants with 0% night wakings.

**Table 1:** Frequency of Night Wakings in Children with Autism Spectrum Disorder

Author(s)	Age range	N	Tool used to measure sleep	Rate of Night Wakings
Rossi et al., 1999	2–20	8	Not specified	44%
Tani et al., 2003	26.5 ± 8.1	20	BNSQ; sleep diary; free description via short essay	30%
Wiggs & Stores, 2004	5–16	69	SPSQ; sleep diary; actigraphy	33%
Williams et al., 2004	2–16	210	Modified sleep survey	34%
Oyane & Bjorvatn, 2005	15–25	15	Sleep diaries; sleep questionnaire; ESS; actigraphy	0%
Ming et al., 2009	3–15	23	Sleep questionnaires; PSG	84.6%
Youssef et al., 2013	4.8–12.8	53	PSG	42%
Ayyash et al., 2015	6.3 ± 1.7 years	9	Sleep diary	31%
Veatch et al., 2016	2–10	80	CSHQ; actigraphy	72%

*BNSQ* Basic Nordic Sleep Questionnaire, *SPSQ* Simonds & Parraga Sleep Questionnaire, *ESS* Epworth Sleepiness Scale, *PSG* = Polysomnography, *CSHQ* Children’s Sleep Habits Questionnaire

### Frequency of Night Wakings in Children with ASD Compared to Other Groups

Table 2 includes the 27 articles in which the prevalence of night wakings was compared between children with ASD and another group, most commonly neuro-typically developing children. Within these, 16 articles reported an increased rate of night wakings compared to other groups, nine articles found no significant difference between the ASD population and the comparison group, and two articles (Anders et al., 2011, 2012) reported lower rates of night wakings in children with ASD compared to other groups.

**Table 2:** Frequency of Night Wakings in Children with ASD Compared with Other Groups

Author(s)	Age range	Sample Size	Tool used to measure sleep	Relationship to Comparison Group
Diomedi et al., 1999	12–24	ASD, n = 10; TD, n = 8	PSG	
Hering et al., 1999	3–12	ASD, n = 8; TD, n = 8	Actigraphy	
Tani et al., 2003	26.5 ± 8.1	ASD, n = 20; TD, n = 10	BNSQ; sleep diary; free description via short essay	
Tani et al., 2004	20 +	ASD, n = 20; TD, n = 10	PSG	
Allik et al., 2006a, 2006b	8.5–12.8	ASD, n = 32; TD, n = 32	Sleep diary; actigraphy; “sleep questionnaire”	
Giannotti et al., 2006	2.6–9.6	ASD, n = 56; TD, n = 56	CSHQ	
Hare et al., 2006	20–58	ASD (+ ID), n = 14; ID, n = 17	Care giver sleep diary	
Hoffman et al., 2006	4–16	autism, n = 106; TD, n = 168	CSHQ	
Bruni et al., 2007	7–15	Asperger, n = 10; autism, n = 12; TD, n = 12	PSG; Bruni questionnaire, PDSS,	
Miano et al., 2007	3.7–19	31	Sleep questionnaire; PSG	
Allik et al., 2008	11.2–15.6	ASD, n = 16; TD, n = 16	Actigraphy	
Giannotti et al., 2008	2–8	ASD, n = 104; TD, n = 162	CSHQ; Sleep diary; 21-channel EEG	
Krakowiak et al., 2008	3.6 years (standard deviation, 0.8 years)	ASD, n = 303; DD, n = 63; TD, n = 163	CHARGE sleep history; CSHQ	
Goldman et al., 2009	4–10	ASD, n = 42; TD n = 16	CSHQ; PCQ; actigraphy; PSG	
Goodlin-Jones et al., 2008	2–5.5	ASD, n = 68; DD, n = 57; TD, n = 69	CSHQ	DD
				TD
Anders et al., 2011	2–5.5	ASD, n = 68; DD, n = 57; TD, n = 69	Actigraphy; sleep–wake diary	DD
				TD
Anders et al., 2012	2–5.5	ASD, n = 69; DD, n = 57; TD = 69	Actigraphy; CSHQ; ESS; sleep diary	DD
				TD
Humphreys et al., 2014	1.5–11	ASD, n = 39; TD, n = 7043	Parent questionnaires	
Baker & Richdale, 2015	21–44	HFASD, n = 36; TD, n = 36	Pittsburgh Sleep Quality Index; sleep wake diary; actigraphy	
Lane et al., 2015	1–6	ASD, n = 68; TD, n = 18;DD, n = 16	Continuous overnight PSG, blood work	DD
				TD
Kheirouri et al., 2016	4–18	ASD, n = 35; TD, n = 31	CSHQ	
Aathira et al., 2017	3–10	ASD, n = 71; TD, n = 65	CSHQ; PSG	
Goldman et al., 2017	11–26	ASD, n = 28; TD, n = 13	ASWS; ASHS; actigraphy; melatonin level; cortisol level	
Benson et al., 2019	18–35	ASD, n = 15; TD, n = 17	Actigraphy; PSQI; STOP-Bang; sleep diary	
Kelmanson, 2020	5	ASD, n = 18; TD, n = 54	CSHQ	
Trickett et al., 2018	2–15	ASD, n = 30; TD, n = 47	SPSQ	
Van der Heijden et al., 2018	6–12	ASD, n = 67; ADHD, n = 44; TD, n = 243	SDSC, parent reported sleep duration	ADHD
				TD

*ASD* Autism Spectrum Disorder, *TD* Typically Developing, *DD* Developmental Delay; *ID* Intellectual Disability, *HFASD* High Functioning Autism Spectrum Disorder, *PSG* polysomnography, *BNSQ* Basic Nordic Sleep Questionnaire, *CSHQ* Children’s Sleep Habits Questionnaire, *PDSS* Pediatric Daytime Sleepiness Scale, *CHARGE* Childhood Autism Risks from Genetics and Environment study, *PCQ* Parental Concerns Questionnaire, *ESS* Epworth Sleepiness Scale, *PSQI* Pittsburgh Sleep Quality Index, *ASWS* Adolescent Sleep Wake Scale, *ASHS* Adolescent Sleep-Hygiene Scale, *STOP-Bang* Snoring, Tiredness, Observed Apnea, Blood Pressure, Body Mass Index, Age, Neck Size, Gender; *SPSQ* Simonds & Parraga Sleep Questionnaire, *SDSC* Sleep Disturbance Scale for Children

Key:—Higher (p > 0.05),—Higher (p < 0.05), —no difference,—Lower (p > 0.05), —Lower (p < 0.05)

#### Evaluation of Tools Used to Identify Night Wakings

Our literature review yielded few articles that objectively evaluated a tool to identify night wakings in clinical practice. Studies comparing multiple tools to validate measurements of night wakings were also limited (Goodlin-Jones et al., 2008; Katz et al., 2018; Malow et al., 2009, 2016; Sitnick et al., 2008). Table 3 summarizes the five studies that utilized validated tools, rating scales, and diagnostic methods to evaluate night wakings. Four of the five studies used the Children’s Sleep Habits Questionnaire (CSHQ) as at least one of the tools to evaluate night wakings. The CSHQ (Owens et al., 2000) is a widely used 45-item validated parent report sleep screening instrument designed to assess sleep disturbance in school aged children. Only one study used actigraphy as compared to polysomnography with video and showed that actigraphy had poor agreement with polysomnography for the detection of night wakings (Sitnick et al., 2008). Three of the five articles in this table employed objective measures of night wakings (actigraphy and/or polysomnography). Notably, across all 76 studies reviewed, most studies relied on parent report or retrospective clinical observation by chart review to report night wakings. The variability in agreement across measures used to identify the occurrence of night wakings underscores the need for a standardized definition and way to identify night wakings in children with ASD.

**Table 3:** Evaluation of Tools Used to Identify Night Wakings

First Author, Year	Age range	ASD Sample Size	Tool used to measure sleep	Results related to Night wakings
Goodlin-Jones et al., 2008	2–5	64	CSHQ; actigraphy;	CSHQ-Night Wakings significantly correlated with actigraphy
Sitnick et al., 2008	2–6	22	Actigraphy; video-somnography	Findings were 94% overall agreement, 97% sensitivity, and 24% specificity. Actigraphy has poor agreement for detecting nocturnal awakenings, compared to video observations
Malow et al., 2009	3–10	93	FISH; CSHQ	The night wakings subscale of the Family Inventory of Sleep Habits was significantly correlated with the CSHQ for TD but not the ASD group (p = .215)
Reed et al., 2009	3–10	20	CGI; CSHQ; actigraphy	Sleep CGI and CSHQ were correlated for night wakings (r = 0.40, p < .001). For each unit increase for CGI-S score, the CSHQ night wakings score increased by 0.647 units. The CGI-S did not show convergent validity with actigraphy measurements of WASO
Katz et al., 2018	4–10	2872	Modified CSHQ for autism;CSHQ	The shorter, modified version of the CSHQ appears useful for identifying night wakings in children with ASD

*CSHQ* Children’s Sleep Habits Questionnaire, *FISH* Family Inventory of Sleep Habits, *CGI* Pediatric Sleep Clinical Global Impressions Scale

### Other Factors or Conditions Associated with Night Wakings

Of the 76 papers reviewed, 21 discussed other factors or conditions associated with night wakings in children with ASD, as shown in Table 4. Medical or developmental co-occurring conditions were grouped into broad categories including developmental / behavioral, neurologic, psychiatric, and medical. Five studies identified night wakings associated with developmental or behavioral issues including anxiety, physical aggression, hyperactivity, hostility inattention, and autism severity (as per Gilliam Autism Rating Scale) (Abel et al, 2018; Giannotti et al., 2008; Kheirouri et al, 2016; Mazurek & Petroski, 2015; Mazurek & Sohl, 2016). Another three reports described neurologic conditions such as greater intellectual disability and developmental regression as associated with sleep disturbance (Taylor et al., 2012; Trickett et al., 2018; Williams et al., 2004). Medical pathology, as expected, was also associated with sleep disturbance. Three studies reported increased rates of night wakings in children with sleep disordered breathing, gastro-esophageal reflux or other gastrointestinal dysfunction (McCue et al, 2017; Tricket et al., 2018; Williams et al., 2004).

**Table 4:** Other Factors or Conditions Associated with Night Wakings

First Author, Year	Age range	ASD Sample Size	Tool used to measure sleep	Associated Condition(s)	Correlation
Honomichl et al., 2002	2–11	100	CSHQ; sleep diary	Parent reported Sleep problems	
				Age	
Williams et al., 2004	2–16	210	Modified sleep survey	Intellectual Disability	
				Vision Problems	
				Respiratory Problems	
				Poor Growth	
Doo & Wing, 2006	2–7.6	193	Chinese version of CSHQ;	Sleep Problem before age 2	
				Age	
Giannotti et al., 2008	2–8	104	CSHQ; Sleep diary; 21-channel EEG	Regressed ASD	
Anders et al., 2012	2–5.5	69	Actigraphy; CSHQ; ESS; sleep diary	PEP-R Perception	
				Eye–Hand Coordination scores	
				PEP-R Fine Motor Coordination	
Goldman et al., 2012	3–18	1859	CSHQ; PCQ	Age	
Taylor et al., 2012	1–18	335	BEDS	Communication Problems	
Richdale et al., 2014	15.5 (1.3)	27	Sleep Diary; SAAQ	SAA-Somatic	
Lane et al., 2015	1–6	68	Continuous overnight PSG, blood work	Serum ferritin levels	
Mazurek & Petroski, 2015	2–18	1347	CSHQ	Age	
				Anxiety	
				Sensory over responsivity	
Kheirouri et al., 2016	4–18	35	CSHQ	Autism Severity	
Malow et al., 2016	4–10	1516	CSHQ; medications taken	Use of sleep medication	
Mazurek & Sohl, 2016	3.6–19.6	81	CSHQ	Physical aggression	
				Hostility	
				Inattention	
				Hyperactivity	
Veatch et al., 2017	2–10	80	CSHQ; actigraphy	Sleep onset delay	
				Age post treatment	
McCue et al., 2017	2–18	610	Medical history data- sleep problems and GI problems	GI problems	
Baker et al., 2017	25 +	28	Actigraphy; sleep diary; saliva collection, melatonin	Night time melatonin concentration	
Abel et al., 2018	2–10	42	Actigraphy	Negative affect	
				Repetitive behaviors	
				CBC	
Benson et al., 2019	18–35	15	Actigraphy; PSQI; STOP-Bang; sleep diary	Next day physical activity	
Kelmanson, 2020	5	18	CSHQ	Affective problems	
Trickett et al., 2018	2–15	30	SPSQ	Sleep Medication Use	
				Language ability	
				GE reflux	
Van der Heijden et al., 2018	6–12	67	SDSC, parent reported sleep duration	Sleep hygiene	

*CSHQ* Children’s Sleep Habits Questionnaire, *ESS* Epworth Sleepiness Scale, *PCQ* Parental Concerns Questionnaire, *BEDS* Behavioral Evaluation of Disorders of Sleep, *SAAQ* Sleep Anticipatory Anxiety Questionnaire, *PSG* Polysomnography, *PSQI* Pittsburgh Sleep Quality Index, *STOP-Bang* Snoring, Tiredness, Observed Apnea, Blood Pressure, Body Mass Index, Age, Neck Size, Gender, *SPSQ* Simonds & Parraga Sleep Questionnaire, *SDSC* Sleep Disturbance Scale for Children, *PEP-R* Psychoeducational Profile Revised

Key:—Positive (p > 0.05), —Positive (p < 0.05),  Negative (p > 0.05),Negative (p < 0.05), -none

Other factors were also reported to be associated with night wakings. Five studies identified an association between child age and night wakings (Doo & Wing, 2006; Goldman et al., 2012; Honomichl et al., 2002; Mazurek & Petroski, 2015; Veatch et al., 2017), with younger children exhibiting more night wakings than older children. One article reported an association between night wakings and next day decreased physical activity (Benson et al., 2019); another reported an association between poor sleep hygiene and increased night wakings (van der Heijden et al., 2018). Examples of poor sleep hygiene included inconsistent sleep and wake times, excessive screen time, screen time in the hours prior to bedtime, and behavioral habits such as drinking a bottle or being rocked to sleep. Ferritin levels were not correlated with wake after sleep onset (Lane et al., 2015). Anders et al (2012) observed an association between sleep disturbance and decreased scores on motor coordination tests.

#### Treatment and Intervention for Night Wakings

Twenty-six of the studies selected addressed treatment of night wakings in individuals diagnosed with ASD, as summarized in Table 5. The majority of these studies (25/26) focused exclusively on a pediatric population (25/26) with only one study including adults between 19 and 52. The sample sizes of the included studies ranged from 2 to 185. Several types of treatment or intervention were used in these studies including medications, parental education, and behavioral interventions. Of the 26 manuscripts assessing treatment options, few were randomized controlled (9/26) studies with the majority using observational methodology (17/26). Of the 26 articles, 23 had evidence graded as Low or Very Low based on the GRADE methodology, three had Moderate evidence, and no articles had evidence rated High.

**Table 5:** Treatment and Intervention for Night Wakings

First Author, Year	Study Type	Age (range or M, SD)	ASD Sample Size	Measure of Night Wakings	Treatment	Effect on Night Wakings	GRADE^85^ Strength of Evidence
Paavonen et al., 2003	Observational	6–17	15	Actigraphy, Parent Reported: CSRF; SDSC	Melatonin		Very Low
Christodulu & Durand, 2004	Observational	2–6	2	Parent Reported: Albany Sleep problems Scale, Parental Sleep Satisfaction Questionnaire	Positive bedtime routines and sleep restriction		Very Low
Garstang & Wallis, 2009	Randomized Controlled Trial	4–16	6	Parent Reported: Sleep Diary	5 mg Melatonin; sleep pamphlet		Low
Giannotti et al., 2006	Observational	2.6–9.6	56	Parent Reported: CSHQ	Controlled-Release Melatonin		Very Low
Ming et al., 2008	Observational	4–16	19	Parent Reported: Sleep diary	Clonidine		Very Low
Wasdell et al., 2008	Randomized controlled trial	2.05–17.81	16	Actigraphy	Controlled-Release Melatonin		Low
Galli-Carminati et al., 2009	Observational	19–52	6	Clinical Observation	Melatonin 3 mg/d– > 6 mg/d– > 9 mg/d		Very Low
Wirojanan et al., 2009	Randomized controlled trial	3–15	8	Actigraphy	Immediate-release Melatonin		Low
Buckley et al., 2011	Observational	2.5–6.9	5	Polysomnography	Donepezil		Very Low
Wright et al., 2011	Randomized Controlled Trial	3–16	16	Parent Reported: Sleep Diary	Melatonin		Low
Adkins et al., 2012	Observational	2–10	36	Actigraphy	Sleep Pamphlet		Low
Cortesi et al., 2012	Randomized Controlled Trial	4–10	185	Actigraphy, Parent reported	1.Controlled-release melatonin		Moderate
					2. Cognitive Behavioral Therapy		
					Combination 1 and 2		
Malow et al., 2012	Observational	3–9	24	Actigraphy, Parent Reported	Melatonin		Very Low
Johnson et al., 2013	Randomized Controlled Trial	2–6	40	Actigraphy	Parent education		Low
Goldman et al., 2014	Observational	3–10	9	Actigraphy, Parent reported	Parent education, Melatonin		Very Low
Knight & Johnson, 2014	Observational	4–5	3	Parent reported	Behavioral treatment package (circadian rhythm management, positive bedtime routines, white noise, graduated extinction)		Very Low
Ayyash et al., 2015	Observational	6.3, 1.7	9	Parent reported	Immediate-release Melatonin (2.5 mg, 5 mg, 10 mg)		Very Low
Stuttard et al., 2015	Observational	5–15	22	Parent Reported: CSHQ	Parent education		Very Low
Loring et al., 2016	Observational	11–18	18	Actigraphy; Parent Reported: ASHS; ASWS; M-ESS	Parent education		Very Low
Veatch et al., 2016	Observational	2–10	80	Actigraphy	Parent education		Low
Frazier et al., 2017	Randomized Control Trial	2.5–12.9	45	Actigraphy, Parent Reported: sleep diary; FISH; CSHQ	Sound-To-Sleep system		Moderate
Gringras et al., 2017	Randomized Controlled Trial	2–17.5	125	Parent reported: SND	Prolonged-Release Melatonin		Low
Narasingharao et al., 2017	Observational	5–16	64	Parent reported	Yoga		Very Low
Maras et al., 2018	Observational	2–17.5	95	Parent reported	Prolonged- release melatonin		Very Low
Mehrazad-Saber et al., 2018	Randomized Controlled Trial	4–16	43	Parent reported: CSHQ	L-Carnosine		Moderate
Delemere & Dounavi, 2018	Observational	2–7	6	Parent Reported: CSHQ	Bedtime fading; Positive routines		Very Low

*CSRF* Children’s self-report form for Sleep Disturbances, *SDSC* Sleep Disturbance Scale for Children, *CSHQ* Children’s Sleep Habits Questionnaire *ASHS* Adolescent Sleep-Hygiene Scale; *ASWS* Adolescent Sleep Wake Scale, *M-ESS* Modified Epworth Sleepiness Scale; *FISH* Family Inventory of Sleep Habits; *SND* Sleep and Nap Diary

Key: —no change, —decrease (p>0.05), —decrease (p<0.05)

Melatonin was the most common medication studied (13/26). Although seven studies showed that melatonin was effective in reducing night wakings (Malow et al., 2012; Galli-Carminati et al., 2009; Giannotti et al., 2006; Garstang & Wallis, 2009; Cortesi et al., 2012; Maras et al., 2018) six studies reported that it did not show significant reduction in night wakings (Ayyash et al., 2015; Gringras et al., 2017; Paavonen et al., 2003; Wasdell et al., 2008; Wirojanan et al., 2009; Wright et al., 2011). Controlled or prolonged release melatonin was evaluated in five of the 26 studies and had variable results. Some studies showed no difference whereas others showed significantly decreased night wakings. Subject groups ranged from 16 to 185 and there were a variety of study types. In the largest of these studies, a randomized controlled trial including 185 subjects (Cortesi et al., 2012), controlled-release melatonin resulted in a significant decrease in night wakings.

Other medications studied included donepezil (Buckley et al., 2011), L- carnosine (Gringras et al., 2017), and clonidine (Ming et al., 2008). The use of donepezil did not result in significant change in night wakings; however, the study had a small sample size (n = 5) and therefore these results were deemed to have Very Low strength (Buckley et al., 2011). Taking L-carnosine also did not result in significant changes in night wakings, a result with Moderate strength evidence (Mehrazad-Saber et al., 2018). Clonidine showed some decrease in night wakings; however, the significance was not assessed and the sample size was small (n = 19), resulting in Very Low strength of evidence for this finding (Ming et al., 2008).

Parent based trainings and behavioral interventions also showed mixed effectiveness across studies. Several studies showed that parent educational trainings, either provided individually or in group settings, were effective in reducing night wakings in children with ASD (Garstang & Wallis, 2009; Goldman et al., 2014; Stuttard et al., 2015; Veatch et al., 2016). Other studies found no significant change in child night wakings due to parent education (Johnson et al., 2013; Loring et al., 2016). Behavioral interventions including cognitive bedtime fading (Christodulu & Durand, 2004; Delemere & Dounavi, 2018), positive routines (Christodulu & Durand, 2004; Delemere & Dounavi, 2018; Knight & Johnson, 2014) and cognitive behavioral therapy (CBT) when used in combination with melatonin were found to decrease night wakings (Cortesi et al., 2012). CBT when used alone did not result in any significant difference in night wakings (Cortesi et al., 2012). Yoga (Narasingharao et al., 2017) was found to significantly decrease night wakings in children with ASD but the Sound-To-Sleep mattress system (Frazier et al., 2017) did not result in a significant change in night wakings in this population.

**Fig. 1:**
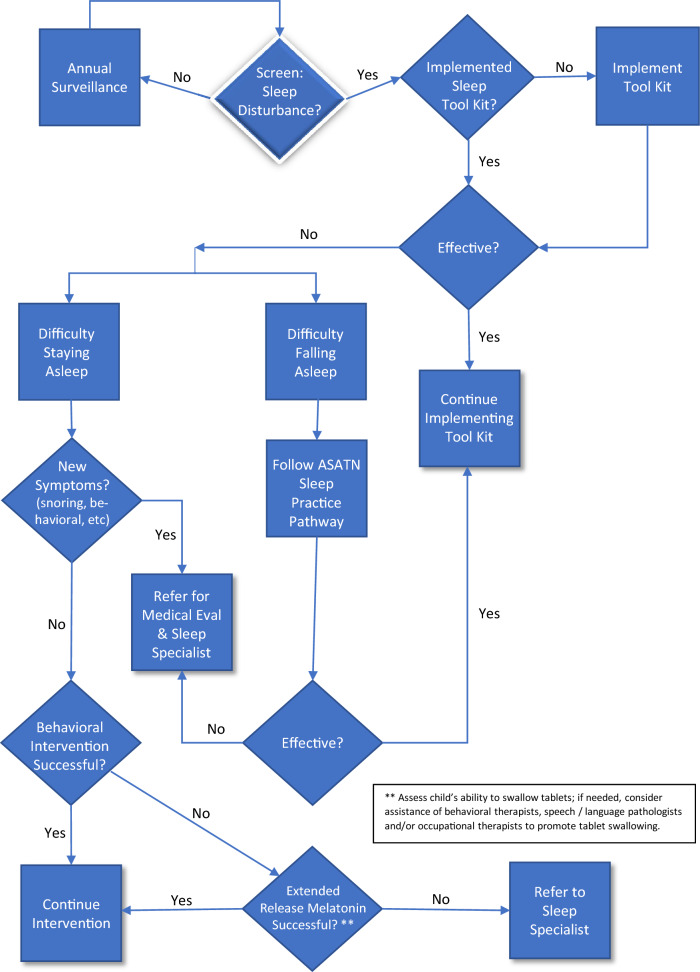
Practice Pathway

Based on the articles reviewed here, we updated the 2012 ATN Sleep Committee’s algorithm for the evaluation and treatment of insomnia in children with ASD using expert consensus within the ATN Sleep Workgroup including physicians and scientists from a variety of pediatric and adult specialties. This updated practice pathway includes the recommendations for night wakings and is the most significant update from the 2012 practice pathway. Figure 1 shows the revised practice pathway that we propose as a guide for practitioners to screen and address their patients’ sleep disturbances. It builds upon the prior pathway proposed in 2012 by the ATN / AIR-P Sleep Workgroup. As in the original pathway, the revised pathway begins with the recommendation for the parent or caregiver to implement the ATN/AIR-P Sleep Toolkit with a child exhibiting sleep disturbance. The Toolkit provides helpful strategies for daytime habits, bedtime rituals and routines, and recommendations for encouraging behaviors and habits that promote sleep. It also includes a “Quick Tips” sheet, visual aids (e.g., colorful printable posters and incentives), and videos. One primary difference between the original pathway and the proposed revised pathway is that the latter distinguishes between children who have difficulty *initiating* sleep vs. those who have difficulty *staying* asleep. The inclusion of night wakings in the revised pathway is justified by the available evidence that at least 34% of children with ASD have night wakings either alone or in combination with delayed sleep onset.”

## Discussion

The vast majority of the articles for this review indicated that children with autism have more frequent or longer night wakings than their neurotypically developing peers. Given the prevalence of sleep problems in children with autism and the significant impact that lack of sleep has on child outcomes, there is alarge need for clinical guidelines to manage sleep disturbances in this population. The Sleep Committee of the ATN / AIR-P set out to update the existing practice pathway on insomnia and autism, as the original version of this practice pathway dealt primarily with sleep onset difficulties. The goal of this article is to provide more specific recommendations on night wakings, a frequent sleep problem within the broader diagnosis of insomnia, i.e., the difficulty to initiate or maintain sleep.

This systematic literature review identified 31 articles that were focused specifically on the identification or treatment of night wakings in individuals diagnosed with ASD, consisting mostly of case reports, small case series, or observational studies. There were few randomized controlled trials found in the published literature addressing night wakings in people with ASD. Due to a lack of randomized controlled trials and small sample sizes, the majority of evidence in available treatment studies was Low or Very Low (23/26) based on GRADE guidelines (Frazier et al., 2017). The paucity of data, especially those stemming from high quality research, in this area was surprising given how prevalent the problem appears.

A further limitation of the studies we reviewed was the apparent lack of a consistent definition for night wakings. A clear and consistent definition of night wakings is not currently available in the literature. The working definition of pathogenic night wakings is based on multiple sources including the ICSD-3 diagnostic criteria for insomnia, definition of an abnormal wake after sleep onset (WASO) from the American Academy of Sleep Medicine Manual for the Scoring of Sleep and Associated Events (Berry et al., 2020). It is also differentiated from physiologic arousals that occur at the end of a sleep cycle and based on available literature about the effects of night wakings on children’s sleep and daytime functioning and the effect on parents and caregivers. Based on our review of the literature, we propose a working definition of “night wakings” to be a waking of 30 min or more following sleep onset, or frequent, greater than 1 per night, shorter night wakings after sleep onset that significantly disrupt the child or their family / caregiver(s). Given the subjective data collected by parental survey and sleep diaries we are unable to clearly define the minimal time necessitated to qualify as a shorter night wakening. Night wakings are noted to be full episodes of arousal, confirmed by polysomnography when available, and exclude parasomnias including but not limited confusional arousals and night terrors. Based on this working definition, we compiled the available literature to update the 2012 practice parameter from this committee as well as to guide our recommendations for further research. This working definition represents a first step in understanding the issue so that we can properly identify the occurrence of night wakings, calculate incidence at different ages, evaluate prevalence accurately, and develop and evaluate efficacious treatment options. Future large population-based samples will be necessary to fully assess the issue. We acknowledge that future modifications to this working definition may be necessary based on future work, which may advance clinical guidance and practice.

With respect to the methods employed to evaluate sleep, objective measures were lacking in most of the studies we reviewed. Notably, objective data gathered via polysomnography and actigraphy did not necessarily validate the subjective data collected from parental reports and validated sleep surveys. Many of the articles reviewed here utilized parent or caregiver reports. Reliance upon parent report of sleep disturbances has been found in both typically developing and atypically developing children to overestimate sleep problems. On the other hand, parental reports are critical to our ability to evaluate and treat sleep disturbances in children; indeed, best practices dictate that caregiver input on child symptoms or problems should be actively sought. The difference between parental report and objective data may represent misperceptions of parents but it may also reflect a higher degree of impact on parental sleep disruption caused by the perception of a higher level of intervention necessary when addressing the night wakings in a child with ASD. The need for a more involved response (e.g., a longer period of wakefulness of the parent while trying to assist the child in returning to sleep) may increase awareness of night wakings by parents who have to intervene. Thus, the impact of night waking may have a more dramatic effect on family and parental function when a greater degree of parental intervention is involved even if the frequency and duration of the actual waking is not different. Conversely, parents of children with ASD may be addressing more worrisome or difficult to manage behaviors or medical issues, leading them to underreport sleep disturbance as it might represent a secondary concern when compared with behavioral or medical issues.

As suggested by the prior practice pathway, we recommend that all children with ASD be screened for sleep problems at their annual visit with their primary provider or more frequently if there are reports of increased difficulty with typical daily activities or academic function. Parents may not necessarily discuss sleep problems during their limited time with a physician or other provider, or the sleep issues may get lower priority compared to other concerns. For these reasons, it is important for providers to query the caregiver for any sleep concerns. Furthermore, given what is known about the impact of night wakings on daytime functioning, we recommend that screening used to identify sleep problems should include queries specific to night wakings. Only four articles since the initial practice pathway addressed tools for screening with sleep questionnaires (CSHQ, CGI, Family Inventory on Sleep Habits), actigraphy, and video-somnography. Questionnaires are the most widely available tool to most clinicians outside of a sleep center, but there are no data indicating how frequently these tools are utilized in routine office visits for children with ASD. Actigraphy is a widely accessible tool and often utilized in adult sleep clinics, but has limitations in its role for identification of night wakings in children with ASD (Sitnick et al., 2008), and the gold standard of polysomnography with video is expensive and not readily available. There is still a need to encourage clinicians and families to discuss sleep problems in the clinical setting so that they can be managed.

The prior practice pathway also emphasized identifying coexisting medical conditions that can contribute to sleep problems. The prior practice pathway also recommended behavioral therapies as a first line approach. Both of these principles are also highlighted in a recent American Academy of Neurology guideline (Williams Buckley et al., 2020). Similar to the articles reviewed from the prior practice pathway on insomnia for ASD, the more recent articles we reviewed provided some evidence that medications may be efficacious in managing night wakings. However, because the findings were inconsistent and sample sizes were often limited, additional information is needed in order to understand the utility of a pharmacological approach to addressing night wakings in this population.

Further research in this area should focus on two main goals:1) better identification of night wakings in larger population studies and 2) evidence-based standards for educational/behavioral and/or pharmacologic interventions to treat night wakings in children with ASD.

Future research needs to define normative values and provide clear descriptors of how night wakings were evaluated in upcoming clinical studies. Objectively measured sleep duration and continuity is currently limited to actigraphy at home which is the less reliable, but most accessible, objective measure for measuring wake after sleep onset in comparison to polysomnography. Video polysomnography can be reliably used to measure night wakings but is currently limited to in-lab polysomnography which is expensive and takes the child out of their typical sleep environment and therefore may not accurately reflect sleep disturbances occurring at home (Penzel et al., 2018). The advent of newly available in-home monitoring with video-time lapse, permitting summary data from sleep patterns at home, may be a new avenue for monitoring and quantifiably identifying sleep disruption as well as assessing efficacy of intervention. Wearable technology for monitoring sleep parameters is becoming more sophisticated and user friendly and warrants further evaluation for efficacy especially with more objective longer-term monitoring. The utility of these monitoring devices is as yet unknown and may be considered in future studies, though data privacy limitations will need to be closely considered.

The identification of other health conditions affecting sleep highlights the need for physicians and providers to do a comprehensive evaluation of the child with sleep disturbance. Since children with decreased intellectual functioning have increased reports of night waking, it also suggests that the medical team and caregiver need to be vigilant about other potential health conditions causing sleep disturbance in a child who may not be able to communicate comparably to neuro-typical peers.

A limitation of the studies reviewed is that most were deemed low or very low on the GRADE system, with only three studies deemed of moderate quality. While the treatment trials reviewed were of low to moderate quality, they do provide some guidance. Randomized controlled trials of medications in children with ASD and insomnia are challenging due to concerns about adverse effects, cost of large studies, need for a reliable and valid outcome measure, and the potential complications of co-occurring medical conditions such as epilepsy, which may be treated with medications that may disrupt sleep. The 2012 Practice Parameter identified the role of melatonin and there is also literature that supports decreased melatonin in children with ASD as compared to their age-matched peers. The literature we reviewed identified similar findings and that extended-release melatonin showed a small to medium effect in the treatment of night wakings for children with ASD. Based on this evidence we included extended-release melatonin as part of the practice parameter. The availability of this formulation of melatonin in tablet form will limit usage in much of this population. Health care provides will need to specifically determine whether a child can swallow tablets. In patients who cannot swallow tablets, health care providers and families may consider the assistance of behavioral therapists, speech/language pathologists, and/or occupational therapists to help promote tablet swallowing. The bulk of the literature suggests the safety and efficacy of melatonin along with recommendations for behavioral and environmental interventions. There was insufficient evidence in the literature to evaluate the use of other interventions or devices (for example, medical safety beds) to address night wakings. However, children with autism may have such profound sleep disturbances so as to compel clinicians to prescribe other sleep medications without evidence-based recommendations. Analysis of existing data may be a starting point in understanding the benefits of medications on sleep since many children with autism already take medications. For example, medications such as clonidine, an alpha agonist sometimes used to treat co-occurring ADHD, may have the dual benefit of treating sleep problems. Untangling these relationships is complex, given that these medications may have variable effects on sleep and sleep architecture. A further consideration in understanding the therapeutic role of medications such as melatonin in addressing sleep disturbances in children with ASD is the timing and dosing amount. There is emerging evidence to suggest that administering a small doe of melatonin several hours prior to bedtime can be beneficial for sleep; however, based on our review of the literature, we felt there was insufficient evidence to support a conclusion about the efficacy of various dose administration strategies and timing.

## Conclusion

The 2012 practice pathway for evaluation and treatment of insomnia in children with ASD is still relevant and continues to be an effective tool for clinicians. The update proposed here provides and estimate of the prevalence of night wakings in youth with ASD and the effects on daytime functioning warrants further study. A clear, universally applied definition of night wakings in future publications is needed. Our working definition is intended to serve as a jumping off point for this work. We define night wakings here as “A waking of 30 min or more following sleep onset, or frequent shorter night wakings after sleep onset that significantly disrupt the child or their family / caregiver.” The etiology of the night wakings may be multi-factorial and this fact may contribute to the difficulty in quantifying these symptoms in children with ASD. We present these data as a tool for practitioners to identify and treat insomnia in children with ASD and to emphasize that further research is necessary.
